# Modified halloysite nanotubes reduce the toxic effects of zearalenone in gestating sows on growth and muscle development of their offsprings

**DOI:** 10.1186/s40104-016-0071-2

**Published:** 2016-02-29

**Authors:** Rui Gao, Qingwei Meng, Jianan Li, Min Liu, Yuanyuan Zhang, Chongpeng Bi, Anshan Shan

**Affiliations:** Institute of Animal Nutrition, Northeast Agricultural University, Harbin, 150030 P. R. China

**Keywords:** Growth, MHNTs, Muscle development, Offsprings, Sows, Zearalenone

## Abstract

**Background:**

Zearalenone (ZEN) is an estrogenic mycotoxin that is primarily produced by Fusarium fungi and has been proven to affect the reproductive capacity of many species to varying degrees. The present experiment was designed to study the maternal persistent effects of zearalenone toxicity in gestating sows on growth and muscle development of their offsprings, and the alleviation of zearalenone toxicity by modified halloysite nanotubes (MHNTs).

**Methods:**

Eighteen sows were fed with one of three dietary treatments that included the following: (1) a control diet, (2) a contaminated grain diet (with 50 % moldy corn, 2.77 mg/kg ZEN), and (3) a contaminated grain diet (with 50 % moldy corn, 2.76 mg/kg ZEN) + 1 % MHNTs. Each sow was exclusively fed its experimental diets from 35 to 70 d of gestation at a total of 2 kg daily. Muscle samples were collected from six piglets per treatment at birth, weaning and finishing.

**Results:**

The results showed that feeding the sows with the ZEN-contaminated diets from 35 to 70 d of gestation decreased the ADG, ADFI and G:F of their offsprings (*P* < 0.05). The muscle fiber numbers in the newborn, weaning and growing-finishing pigs and the muscle fiber diameters at birth and weaning were also decreased by maternal ZEN exposure (*P* < 0.05). The expressions of *IGF-I*, *IGF-II*, *Myf-5* and *Mstn* at birth and *IGF-II*, *Pax7*, *Myf-5* and *MyoD1* at weaning were altered by feeding gestating sows with ZEN-contaminated diets (*P* < 0.05). The MHNTs reduced most of the ZEN-induced toxic effects: the ADG and ADFI on growth performance, the muscle fiber numbers at weaning and finishing and the muscle fiber diameters at weaning (*P* < 0.05). The expression levels of *IGF-II* and *Mstn* in newborn piglets and *IGF-II* and *Myf-5* in weaning piglets were also prevented by adding 1 % MHNTs (*P* < 0.05).

**Conclusions:**

The present study demonstrated that the offsprings of sows fed with ZEN-contaminated diets from 35 to 70 day of gestation exhibited weakening on growth performance, physiological changes in their muscle fibers and alterations of mRNA expression in their muscle tissues, and also indicated that MHNTs prevented most of the ZEN-induced weakening in the muscle tissues.

## Background

Fusarium graminearum is most frequently isolated from maize in temperate climates, and zearalenone production often occurs during the cold weather storage of high-moisture feeds carrying the mold [1]. Zearalenone (ZEN) is a mycotoxin that is produced primarily by fungi of the genus Fusarium in foods and feeds. ZEN has frequently been implicated in reproductive disorders in farm animals and occasionally in hyperoestrogenic syndromes in humans [2]. In farm animals, swine seem to be particularly sensitive to mycotoxins [3]. Meanwhile, maternal effects which play an important role on offspring’s growth and muscle development were proved by many previous researchers [4]. ZEN-related problems frequently occur in piglets under natural conditions and are by exposure in the uterus, the placental transfer from an exposed sow to her piglets and by ZEN stored in the sow during gestation and released via the suckling of the piglets [5].

There has been an increased interest in the application of halloysite nanotubes (HNTs) in order to find a way to detoxify contaminated feedstuffs or diets which is appropriate for large quantities of raw material sources, inexpensive, simple and results in products of stable quality. Halloysite is a type of aluminosilicate clay with a hollow nano-tubular structure and a set of characteristics that make it cheap, abundantly available, durable, highly mechanically strong and biocompatible [6]. Around the world, halloysite nanotubes have been used as nanocomposites, nanocontainers [7] and new drug carriers in medicine [8, 9], but the use of HNTs in animals as adsorbents has not yet been reported.

Indeed, many studies of laboratory animals, farm animals and humans have reported that exposure to the estrogenic effects of ZEN causes relevant reproductive performance alterations [10, 11]. It has been reported that maternal zearalenone exposure causes fetal malformations and physiological alterations to sexual organs [12, 13]. However, few studies have examined the effects of zearalenone on growth or muscle development, particularly in combination with maternal effects. These factors prompted us to begin a more extensive investigation of the possible effects of maternal zearalenone exposure on offspring growth and muscle development and to exploit a new adsorbent to mitigate the negative effects of zearalenone contamination.

## Methods

### Mold strain

Fusarium graminearum has previously been shown to produce ZEN in corn [14]. The fungus used in this experiment was purchased from the Agricultural Culture Collection of China (No. ACCC36249) and cultivated on potato dextrose agar (PDA, potato extract 0.4 %, glucose 2 % and agar 1.5 %, pH 5.6 ± 0.2). The experimental culture media were obtained from Fluka (Bornem, Belgium) [15].

The corn used in this experiment was obtained from Xiang Fang Experimental Bases (Northeast Agricultural University, China) and milled in a hammer mill with a 40-mesh screen (Trapp-TRF model 90). The in vitro ZEN production was conducted according to the procedures outlined by Lígia Martins [14]. The studies of mycotoxin production by Fusarium graminearum were performed in duplicate on trays containing 1,000 g of sterilized cracked corn following the addition of 400 mL of distilled water and adjusting the aw to 0.98 by water activity meter (HD-4, HuaKe Instrument & Meter Co. Ltd., Wuxi, China).

The corn contained no fungal infection or ZEN contamination. Autoclaved substrate was inoculated with 40 mL of the spore suspension according to the following procedure: 100 mL of sterile distilled water were added to each slant of the 5-day-old culture, and the surface of the agar was gently scraped to produce a turbid suspension with 1 × 10^14^ spores/mL. One hundred milliliters of this suspension were added to the cracked corn. The inoculated flasks were stirred daily for the first 5 d. The culture conditions used in this experiment, 28 °C for 15 d followed by 12 °C with ZEN peaking on the 35th day of incubation, reflected the earlier work reported by Lígia Martins [14]. To equalize the moisture contents of the samples, each sample was dried at 60 °C for 96 h and stored in a freezer at −20 °C until analysis [16].

### Modification of the adsorbent

The adsorbent used in experiment was that described by Zhang et al. [17]. Halloysite nanotube powder (HNTs), was purchased and refined from Golden Sunshine Ceramics Co., Ltd. (Zhengzhou, China). The molecular formula of halloysite nanotube was Al_2_Si_2_O_5_(OH)_4_ · nH_2_O, composed of 95 % kaolinite, trace amounts of quartz and natroalunite. The crystal diameter was 10 to 50 nm and the crystal length was less than 1 μm. The proportion of granule (<2 μm) was 70 % in the original mineral.

The previously reported method of Jinhua was used to prepare the powder [7]. All solutions were prepared using distilled water. The powder was prepared as follows: A water suspension solution (5 % in mass) was prepared by adding water to dry halloysite mineral. The suspension solution was intensively stirred for 2 h and sprayed to dry at 200 °C to obtain fine powder. Before use, dry halloysite powder was sieved to eliminate aggregates.

Modification of the halloysite nanotubes was accomplished using stearyldimethylbenzylammonium chloride (SKC) as a surfactant (Jingwei Chemical Co., Ltd., Shanghai, China) according the previously described methods of Lemke [18]. HNT (100 g) were treated with 1,000 mL distilled water containing SKC (0.5 %) and mixed at a speed of 2,300 g with a reaction time of 10 min at 50 °C. When the reaction was complete, the suspensions were filtered, washed three times with deionised water, dried at 80 °C and crush 100 g of HNT to obtain particles that were less than 45 μm in a beater mill at 7,400 g for 3 min. The made-up particles were added in the contaminated diets evenly to make the MHNTs diets.

**Fig. 1:**
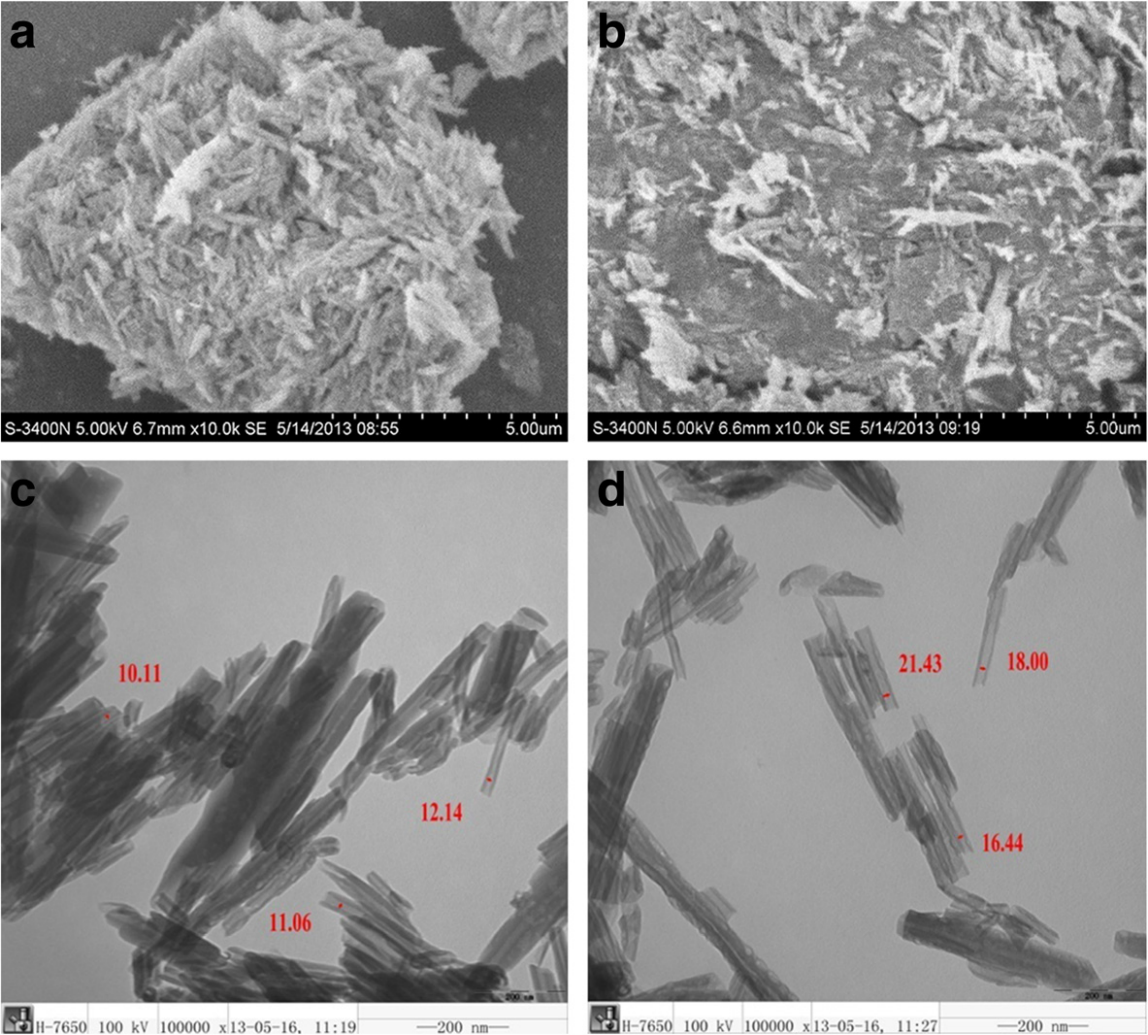
**a** Scanning electron microscope (EM) image of the halloysite nanotubes (HNT) powder. **b** Scanning EM image of modified HNT (MHNTs) powder with increased dispersity. **c** Transmission EM images of the HNT. **d** Transmission EM image of modified HNT with enlarged internal diameters [17]

Figure 1 shows the images after electric microscope (EM). It can be seen that the samples consisted of cylindrical tubes. After treatment with SKC, the dispersion of the halloysite (Fig. 1b) was increased compared to the untreated HNT powder (Fig. 1a). Moreover, the lumen of modified nanotubes shown in Fig. 1d was enlarged compared with untreated nanotubes (Fig. 1c). As shown in Fig. 1c and d, the external surface of the treated HNT was cruder than the natural halloysite [17].

### Animals and experimental design

Eighteen pregnant Yorkshire sows were randomly divided into the following three treatment groups (6 per treatment): (1) control, (2) contaminated grains (with 50 % moldy corn); and (3) contaminated grains (with 50 % moldy corn) + 1 % modified HNTs (MHNTs). The doses of the MHNTs were selected based on the research of Jiang [19, 20]. The pregnant sows were housed in individual stalls after 35 d of gestation (GDs). Each sow was fed exclusively with the appropriate experimental diet from d 35 to d 70 of gestation at a total of 2 kg daily. All of the feedstuffs were subjected to post-processing analytical control. The feed compositions were compared using validated analytical methods (National Standards of the People’s Republic of China, GB/T 19540–2004). ZEN was the major contaminant and was present at 0.03 mg/kg in the control diet, 2.77 mg/kg in the contaminated diet, 2.76 mg/kg in the adding adsorbent contaminated diet. The concentration of deoxynivalenol (DON) which was also produced by Fusarium graminearum was 0.04 mg/kg in the contaminated diet, lower than the limits of national standards (1 mg/kg). Analyses of the corn and diets with GC-MS were performed to provide detailed characterizations of the trichothecene mycotoxin patterns and revealed that the B-trichothecene mycotoxins, such as 15-acetyldeoxynivalenol, 3-acetyldeoxynivalenol, and nivalenol, and A-trichothecene mycotoxin HT-2 toxins were lower than the detection limits [21]. At 7 d of age all piglets, the piglets received an iron injection, and the males were castrated. The piglets were weaned at 21 d of age and moved to post-weaning rooms with an ambient temperature of 27 °C. After weaning, the piglets had ad libitum access to the standard diets and water intake until finishing in individual pens. All experimental diets (Table 1) were formulated to meet or exceed the National Research Council nutrient requirements (2012) [22].

**Table 1:** Percentage composition of the diet

Parameters	Control^c^	Contaminated grains^c^	Contaminated grains + 1 % MHNTs^c^	Lactation
Ingredient, %				
Control corn	62.40	31.20	31.20	63.80
Contaminated corn	-	31.20	31.20	-
Soybean meal	16.00	16.00	16.00	20.00
Wheat bran	18.00	18.00	17.00	-
MHNTs	-	-	1.00	-
Full-fat soybean	-	-	-	12
Limestone	1.00	1.00	1.00	0.93
Dicalcium phosphate	1.10	1.10	1.10	1.77
Salt	0.50	0.50	0.50	0.50
Vitamin and mineral premix^a^	1.00	1.00	1.00	1.00
Analyzed composition				
Metabolizable energy, MJ/kg^a^	11.90	11.84	11.75	12.90
Crude protein	14.51	14.45	14.31	18.48
Calcium	0.69	0.68	0.67	0.79
Total phosphorus	0.61	0.61	0.60	0.64
Lysine	0.65	0.67	0.66	0.98
Tryptophan	0.16	0.16	0.16	0.23
Threonine	0.52	0.52	0.52	0.69
Methionine + Cystine	0.39	0.45	0.45	0.52
Concentration of ZEN^c^, mg/kg	0.03	2.77	2.76	0.01

^a^Provided the following per kilogram of diet: Cu, 18.2 mg; Zn, 126.0 mg; Se, 0.3 mg; Mn, 50.5 mg; Fe, 150.3 mg; I, 0.4 mg; vitamin A, 11, 050 IU; vitamin D, 2,310 IU; vitamin E, 62.8 IU; vitamin K, 2.6 mg; riboflavin, 5.8 mg; pantothenic acid, 20 mg; niacin, 25 mg; vitamin B12, 326 μg; folate, 6.5 mg; pyridoxine, 1.8 mg; biotin, 350 μg; and thiamin, 1.9 mg

^b^Calculated values according to the Tables of Feed Composition and Nutritive Values in China [42] in this study

^c^Control: control diet; Contaminated grains: instead of 50 % moldy corn; Contaminated grains: instead of 50 % moldy corn + 1 % MHNTs; *ZEN* Zearalenone, *MHNTs* modified Halloysite nanotubes

### Sample collection

All of the animal experimental procedures were approved by the Ethical and Animal Welfare Committee of Heilongjiang Province, China.

Six piglets in each group were slaughtered via an intraarterial injection of pentobarbital (200 mg/kg) for general anesthesia at birth and six more at weaning. At the end of the experiment, 18 growing-finishing pigs from the three treatments were transported to the abattoir for slaughter. At birth, weaning and finishing, one pig was selected from each litter and slaughtered to collect samples.

After slaughter, longissimus muscle samples were quickly collected, frozen in liquid nitrogen, stored at −80 °C and analyzed for gene expression by RNA extraction, followed by quantitative reverse transcription PCR [11]. Portions of the longissimus muscle samples were fixed in 4 % paraformaldehyde in phosphate buffer (0.12 mol/L, pH 7.4) for histochemical examination. The remaining organs were directly stored at −20 °C for analysis.

### Growth performance

The growth performance was evaluated from weaning to finishing. All the pigs were weighed individually at weaning and slaughter. The feed intakes per pen were recorded after weaning. The average daily gain (ADG), average daily feed intake (ADFI) and gain/feed (G:F) values were calculated.

### Histochemical examination

The longissimus muscle samples were embedded in paraffin and cut into 10-μm sections. The muscle sections were rehydrated via a series of incubations in xylene and ethanol solutions and then stained with hematoxylin and eosin for standard light microscopy. Ten fields were randomly selected to quantify the muscle fiber diameters. The majority of the muscle fibers were circular; thus, the diameters were easily measured. For the irregular muscle fibers, the maximum and minimum diameters of the muscle fiber circle were measured, and the average value of the maximum and minimum was regarded as the diameter of the fiber. The diameters of 10 muscle fiber per field were measured, and 100 muscle fibers per sample were quantified using the Motic Images Plus 2.0 software. The muscle fiber diameters were measured in a blinded fashion. The averaged data were used for calculations [4]. The percentages of muscle fibers were calculated based on the total fiber numbers per examined area, and this latter value for the control treatment was regarded as 100 % [23].

### Quantitative real-time PCR

Total RNA was extracted from the muscles (30 mg of tissue) of the piglets using the Trizol reagent (E.Z.N.A. ® Total RNA Kit, Omega Bio-tek, Inc., United States). The RNA concentration was measured with a spectrophotometer at 260/280 nm. The quality of the RNA was estimated by detecting the number of bands by agarose gel electrophoresis.

SYBR green I real-time polymerase chain reactions (RT-PCR) were used to measure the mRNA expression of *IGF-I*, *IGF-II*, *Pax7*, *Myf-5*, *MyoD1*, *Mstn* and *β-actin*. First-strand cDNA was synthesized from 5 μg of total RNA (processed using DNase) using oligo (dT) primers and Superscript II reverse transcriptase according to the manufacturer’s instructions (Tiangen Biotech Co., Ltd, Beijing, China). Real-time PCR was performed in an ABI PRISM 7500 SDS thermal cycler (Applied Biosystems, Foster City, CA). Each sample was analyzed in triplicate. The primers used in the analyses are listed in Table 2. The reactions were performed with 2.0 μL of first-strand cDNA and 0.8 μL of sense and anti-sense primers in a final volume of 20 μL as recommended by the SYBR real-time PCR kit (TaKaRa® BIO CATALOG, Da Lian, China). The RT-PCR conditions were as follows: 1 cycle at 95 °C for 30 s, and 40 cycles at 95 °C for 5 s and 60 °C for 34 s. The relative expressions of the inflammatory cytokine mRNAs were determined with the 2^-ΔCt^ method [24].

**Table 2:** Primers used for quantitative real-time PCR

Gene	GenBank Accession number	Primer sequence (5’→3’)^a^	Amplicon length, bp
*β-actin*	AY_550069	FP: ATGCTTCTAGGCGGACTGT RP: CCATCCAACCGACTGCT	211
*IGF-I*	NM_214256	FP: CTGTGCTTGCTCTCCTTCAC RP: TACCCTGTGGGCTTGTTGA	128
*IGF-II*	NM_213883	FP: GTGGCATCGTGGAAGAGTG RP: GTGGCATCGTGGAAGAGTG	166
*Pax7*	XM_005659088	FP: CCACATCCGCCACAAGATAG RP: ATGCCTGGGTTCTCCCTCT	162
*Myf-5*	NM_001278775	FP: CCAGCCTCTCTCTCTCCAGTT RP: GCCTCCTTCCTCCTGTGTAATA	151
*MyoD1*	NM_001002824	FP: AGCGGACGACTTCTATGATGAC RP: GTGTTCCTCGGGCTTTAGG	112
*Mstn*	NM_001012406	FP: TGGTATTTGGCAGAGCATTGAT RP: CCTGGGAAGGTTACAGCAAGAT	129

^a^ *FP* forward primer, *RP* reverse primer

### Statistical analyses

All data were analyzed with SPSS software (SPSS Inc., Chicago, IL, USA) and the results were expressed as LSMEANS, SEM and *P*-values. When treatment differences were detected by ANOVA, the significance of the differences between the treatments was determined with Duncan’s multiple range tests. Significance was considered at the probability level of *P* < 0.05.

## Results

### Growth performance

The growth performance of offsprings are presented in Table 3. The average daily gain and the average daily feed intake decreased in the corn contaminated with mold group compared with the control group (*P* < 0.05). The gain/feed was also decreased by maternal zearalenone exposure (*P* < 0.05).

**Table 3:** Growth performance

Item	Control	2.77 mg/kg ZEN	2.76 mg/kg ZEN+ 1 % MHNTs
ADG, kg/d	0.68 ± 0.01^a^	0.61 ± 0.01^c^	0.64 ± 0.01^b^
ADFI, kg/d	1.88 ± 0.03^a^	1.77 ± 0.03^b^	1.86 ± 0.03^a^
G:F	0.36 ± 0.01^a^	0.34 ± 0.01^b^	0.35 ± 0.01^ab^

Data are means ± SEM

*ZEN* zearalenone

*MHNTs* modified halloysite nanotubes

*ADG* average daily gain, *ADFI* average daily feed intake, *G*:*F* gain/feed

^a, b, c^means within a row with no common superscripts differ significantly (*P* < 0.05)

The average daily gain raised by adding 1 % MHNTs to maternal diets (*P* < 0.05), but the level did not reach the ADG of control animals. The average daily feed intake also raised following the addition of 1 % MHNTs to the diets (*P* < 0.05).

### Muscle fiber diameters and numbers

The muscle fiber diameters and numbers of offsprings are presented in Table 4. The muscle fiber numbers in the newborn, weaning and growing-finishing pigs were decreased in ZEN-treated group compared with the control group (*P* < 0.05). The moldy corn group exhibited the lower muscle fiber diameters at birth and weaning compared to the control group (*P* < 0.05). There were no differences in the muscle fiber diameters of finishing pigs between the three treatments.

**Table 4:** Muscle fiber diameters and numbers

Item	Control	2.77 mg/kg ZEN	2.76 mg/kg ZEN+ 1 % MHNTs
Newborn			
Muscle fibre numbers	1.00 ± 0.02^b^	0.93 ± 0.02^a^	0.95 ± 0.02^ab^
Muscle fibre diameter	9.98 ± 0.42^b^	8.40 ± 0.33^a^	9.48 ± 0.40^ab^
Weaning			
Muscle fibre numbers	1.00 ± 0.02^b^	0.93 ± 0.03^a^	1.01 ± 0.01^b^
Muscle fibre diameter	18.55 ± 0.86^b^	15.40 ± 0.79^a^	18.78 ± 0.96^b^
Growing-finishing			
Muscle fibre numbers	1.00 ± 0.01^b^	0.97 ± 0.01^a^	1.00 ± 0.01^b^
Muscle fibre diameter	49.55 ± 1.63	49.12 ± 1.04	48.78 ± 1.64

Data are means ± SEM

*ZEN* zearalenone

*MHNTs* modified halloysite nanotubes

^a, b, c^means within a row with no common superscripts differ significantly (*P* < 0.05)

Muscle fiber numbers and diameters at birth, though higher compared to moldy corn group, do not differ significantly when MHNTs were added to maternal diets. Nevertheless, following the addition of MHNTs to maternal diets muscle fiber numbers increased at weaning and slaughter up to control animals levels (*P* < 0.05), and MHNTs reduced the damage in muscle fiber diameters at weaning (*P* < 0.05).

### The mRNA expression in the longissimus muscle

The mRNA expressions of *IGF-I*, *IGF-II*, *Pax7*, *Myf-5*, *MyoD1* and *Mstn* in the longissimus muscle are presented in Table 5. The mRNA expression of *IGF-I* was decreased in the ZEN-contaminated group at birth (*P* < 0.05). No differences in the mRNA expression of *IGF-I* were observed between the ZEN-contaminated group and the control group at weaning. Maternal zearalenone exposure reduced the expression of *IGF-II* both at birth and weaning (*P* < 0.05). The results for *Myf-5* and *MyoD1* were similar: the expression of *Myf-5* was reduced at birth and weaning (*P* < 0.05). The expression of *MyoD1* was also reduced at weaning (*P* < 0.05), but not at birth (*P* > 0.05). The expression of *Pax7 *was not affected by zearalenone at birth (*P* > 0.05) but reduced at weaning (*P* < 0.05). Maternal zearalenone exposure increased the expression of *Mstn* at birth (*P* < 0.05) but not at weaning (*P* > 0.05). However, there were no differences on the mRNA expressions of *IGF-I*, *IGF-II*, *Pax7*, *Myf-5*, *MyoD1* and *Mstn* in finishing pigs between ZEN-contaminated group and the control.

**Table 5:** The mRNA expression in the longissimus muscle

Item	Control	2.77 mg/kg ZEN	2.76 mg/kg ZEN+ 1 % MHNTs
Newborn			
*IGF-I*	0.0219 ± 0.0020^b^	0.0086 ± 0.0006^a^	0.0092 ± 0.0004^a^
*IGF-II*	3.2737 ± 0.2822^b^	1.4926 ± 0.0674^a^	3.1937 ± 0.1827^b^
*Pax7*	0.0076 ± 0.0010	0.0063 ± 0.0006	0.0059 ± 0.0007
*Myf-5*	0.0220 ± 0.0030^b^	0.0115 ± 0.0014^a^	0.0111 ± 0.0015^a^
*MyoD1*	0.0176 ± 0.0020	0.0134 ± 0.0008	0.0134 ± 0.0016
*Mstn*	0.0023 ± 0.0002^b^	0.0032 ± 0.0002^a^	0.0020 ± 0.0001^b^
Weaning			
*IGF-I*	0.0138 ± 0.0037	0.0094 ± 0.0011	0.0138 ± 0.0022
*IGF-II*	6.8816 ± 0.4270^b^	2.4085 ± 0.1870^a^	5.5329 ± 0.3988^c^
*Pax7*	0.0056 ± 0.0009^b^	0.0031 ± 0.0004^a^	0.0045 ± 0.0006^ab^
* Myf-5*	0.0170 ± 0.0032^b^	0.0040 ± 0.0004^a^	0.0107 ± 0.0021^b^
*MyoD1*	0.0263 ± 0.0038^b^	0.0101 ± 0.0021^a^	0.0152 ± 0.0045^a^
*Mstn*	0.0027 ± 0.0006	0.0038 ± 0.0002	0.0029 ± 0.0002
Growing-finishing			
*IGF-I*	0.1073 ± 0.0234	0.0899 ± 0.0160	0.1288 ± 0.0219
*IGF-II*	6.1416 ± 0.4666	5.3906 ± 0.4628	4.8610 ± 0.5954
*Pax7*	0.0232 ± 0.0042	0.0173 ± 0.0048	0.0220 ± 0.0023
*Myf-5*	0.0212 ± 0.0011	0.0302 ± 0.0052	0.0246 ± 0.0049
*MyoD1*	0.2534 ± 0.0184	0.3023 ± 0.0254	0.2642 ± 0.0322
*Mstn*	0.0336 ± 0.0037	0.0341 ± 0.0032	0.0371 ± 0.0028

Data are means ± SEM

*ZEN* zearalenone

*MHNTs* modified halloysite nanotubes

^a, b, c^means within a row with no common superscripts differ significantly (*P* < 0.05)

In this experiment, MHNTs reduced the toxic effects on the expressions of* IGF-II* and *Mstn* in newborn piglets (*P* < 0.05), the expressions of *IGF-II* and *Myf-5* (*P* < 0.05) in weaning piglets. The results might show a tendency in reduction of toxic effects on expression of *IGF-I,Pax7*, *MyoD1* in weaning piglets and expressions of* IGF-I* and *Pax7* in growing-finishing pigs, but the mean values obtained do not differ statistically significantly from those obtained for animals fed the ZEN-contaminated diet.

## Discussion

Because the nutritional levels of the three treatments during gestation were similar, and the other experimental conditions were also identical, we hypothesize that the changes in the muscle fibers and mRNA expressions in the offsprings were predominantly caused by maternal ZEN exposure. The ameliorative effects of the treatment with MHNTs were considered to be the result of the addition of this adsorbent to the diet in our study.

The researches on growth performance and muscle development of zearalenone were lacking. Our previous research demonstrated that maternal zearalenone exposure in gestating sows decreased the average body weight at birth and weaning of piglets, and the average daily gain of weaning piglets were also reduced [25]. The previous results were in accord with this experiment. Doll et al. [26] also observed that feed intake and growth rates were reduced in gilts fed diets with deoxynivalenol and zearalenone (DON, 8.6 mg/kg; ZEN, 1.2 mg/kg), but the toxin which took effects primarily were undefined. However, some researches had the different conclusions. Zearalenone had no effects on growth performance in prepubertal gilts [27]. Jiang et al. [19] observed gilts fed different amounts of dietary ZEN grew similarly with no differences in ADG and ADFI.

The growth and development of skeletal muscle includes the increases in muscle fiber numbers (hyperplasia) and the enlargements in the volumes of the muscle fibers (hypertrophy). The number of muscle fibers is set a birth, and no further increases occur. The growth and development of muscle consists of only increases in the volumes of muscle fibers; thus, the fetal period plays a key in skeletal muscle development [28, 29]. In the embryo, a spot of muscle fibers (primary myofibers) begins to develop, and the majority of muscle fibers (secondary myofibers) are formed in the fetal period. The primary myofibers of pigs are formed during the 38 d of early gestation. The secondary myofibers are formed from 46 to 95 d of gestation, and muscle fiber numbers do not increase after this period [30, 31]. Skeletal muscle lies at the bottom end of nutrient partitioning during development. As nutrient substances are always first assigned to the nervous system, organs and skeleton, skeletal muscle can easily be affected by maternal nutrition fluctuations [4]. Therefore, such fluctuations can cause irreversible decreases in the numbers of muscle fibers in the fetal period. In the present experiment, maternal ZEN exposure decreased the muscle fiber numbers permanently, muscle fiber diameter were also reduced at birth and weaning compared with the control group. These results accorded with our previous research that the average body weight (BW) of fetuses at 70 d in pregnant, the litter birth weight, the average BW of piglet, and the born alive piglet BW at farrowing were all decreased by ZEN exposure [25]. In contrast to the current study, Kiessling et al. [32] reported that no significant changes in fiber number or diameter occur in male rats after prolonged zearalenone (1.25 or 3.75 mg/kg) feeding. ZEN has been found to be maternally toxic and fetotoxic but not teratogenic [33], and it seems that ZEN is unable to affect muscle fibers that have already formed in adult animals. Obviously, the fetuses were affected by maternal toxicity because the sows were fed contaminated diets from 35 to 70 d of gestation, which is the key period of secondary myofiber development. Differences in the muscle fiber diameters between the three treatments were not observed in the growing-finishing pigs, and these results indicate that the compensation abilities of the offsprings were able to partially eliminate the previous differences.

The process of muscle formation is regulated by series of transcription factors in the embryo that include Wnt, paired boxes (Pax3 and Pax7) and myogenic regulatory factors (MRFs). Pax3 and Pax7 can induce the expression of myogenic regulatory factors and transform myogenous cells to myoblasts [34, 35]. Several myogenic regulatory factors had been found thus far including myogenic differentiation (MyoD), myogenic factor 5 (Myf-5), myogenin and myogenic factor 4 (Myf-4). The actions of MyoD and Myf-5 induce multifunctional myogenous cells to differentiate into myoblasts, and the functions of MyoD and Myf-5 seem to be complementary [34, 36]. In vitro results have indicated that myostatin (Mstn) can directly affect the proliferation or differentiation of many skeletal muscle cell systems and that Mstn can regulate the differentiation of myoblasts by down-regulating the myogenic differentiation factors MyoD, Myf-5 and myogenin [37]. In our experiment, the expression of *Pax7*, *Myf-5* and *MyoD1* were reduced at different periods by maternal ZEN exposure. Meanwhile, the increased expression of *MSTN* also accorded with our results of muscle fiber deterioration. Zhang et al. [25] indicated that placenta weight and the apoptosis-related mRNA expression were altered in the ZEN treatment groups in the placenta and uterus of the sows and piglets. The pathologic uterine and placental changes could affect the functions of the organs and the transportation of nutrition to the fetuses, which may explain the adverse effects on the fetuses [25]. We observed that the expressions of *MyoD* and *Myf-5* were increased by ZEN-contaminated treatment at finishing, although these differences were not significant. It is possible that the offsprings in the control group had developed completely, and the slower development of the offsprings in ZEN group had begun to be offset by the compensatory processes. IGFs play critical roles in skeletal muscle differentiation and growth, and previous studies have shown that IGFs not only stimulate myoblast proliferation but also promote myogenic differentiation, which are two mutually exclusive processes [38]. In the present study, the mRNA expression of *IGF-I* and * IGF-II* were both decreased in the ZEN-contaminated group at different periods. Previous research has shown that no changes on the expressions of IGF-I or IGF-II was observed by using a diet contaminated with fusarium (4.42 mg/kg DON, 0.048 mg/kg ZEN) from 35 to 70 d of gestation in neither sows or offsprings compared to a control diet [21]. Oliver et al. [27] observed that zearalenone (1.5 mg/kg) does not alter skeletal muscle signaling in prepubertal gilts. The previous research seems to indicate that zearalenone might affect reproductive performance and thereby affect the development of the offsprings and that ZEN might not induce physiological changes in the muscle fibers or skeletal muscle signaling within a generation. However, our results proved that ZEN has a trans-generational toxicity that affected the muscle development of the offsprings.

With the use of an adsorbent modified with the surfactant SKC, the harmful effects of hydrophobic pesticides from a pollution source can be prevented by clay and soil [39], and the ZEN adsorption efficacies of HNTs and MHNTs have been shown in our previous research [17]. However, the inherent safety of halloysite should also be concerned. The results of Lai et al. [40] indicate that halloysite exhibits a high degree of biocompatibility characterized by an absence of cytotoxicity, in spite of elevated pro-inflammatory cytokine release. Halloysite nanotubes were also found safe for *C. elegans* at a concentration up to 1mg/mL  which is about 1,000 times higher than the possible soil contamination concentrations, therefore its quickly growing industrial application is likely to be environmentally safe [41]. In the present experiment, MHNTs alleviated the toxic effects of ZEN on muscle fiber diameters and numbers in piglets. MHNTs also increased the expressions of *IGF-I*,* IGF-II*, *Pax7*, *Myf-5* and *MyoD1* and reduced the expression of Mstn; these factors are closely related to the physiological changes that occur in muscle fiber. Although the MHNTs did not restore the partial indexes of the offsprings to the normal levels of the control group, an alleviative tendency was observed relative to the ZEN-treated group.

## Conclusions

The present study demonstrated that the offsprings of sows fed with ZEN-contaminated diets from 35 to 70 d of gestation exhibited weakening on growth performance, physiological changes in their muscle fibers and alterations of mRNA expression in their muscle tissues. Our results also indicated that MHNTs prevented most of the ZEN-induced weakening on growth performance, physiological changes in the muscle fiber and the alterations of mRNA expression in the muscle tissues. MHNTs might be used as effective adsorbents in the feed during the production of sows to alleviate damage to the progeny.

## Abbreviations

ZEN: Zearalenone
MHNTs: Modified halloysite nanotubes
ADG: Average daily gain
ADFI: Average daily feed intake
G:F: gain/feed
